# Enhancing Stability and Performance in Mobile Robot Path Planning with PMR-Dueling DQN Algorithm

**DOI:** 10.3390/s24051523

**Published:** 2024-02-27

**Authors:** Demelash Abiye Deguale, Lingli Yu, Melikamu Liyih Sinishaw, Keyi Li

**Affiliations:** 1 School of Automation, Central South University, Changsha 410083, China; llyu@csu.edu.cn (L.Y.); 214612147@csu.edu.cn (K.L.); 2 School of Computer Science and Engineering, Central South University, Changsha 410083, China; melikamuliyih29@gmail.com

**Keywords:** path planning, dueling network, mobile robot, prioritized experience replay, reinforcement learning

## Abstract

Path planning for mobile robots in complex circumstances is still a challenging issue. This work introduces an improved deep reinforcement learning strategy for robot navigation that combines dueling architecture, Prioritized Experience Replay, and shaped Rewards. In a grid world and two Gazebo simulation environments with static and dynamic obstacles, the Dueling Deep Q-Network with Modified Rewards and Prioritized Experience Replay (PMR-Dueling DQN) algorithm is compared against Q-learning, DQN, and DDQN in terms of path optimality, collision avoidance, and learning speed. To encourage the best routes, the shaped Reward function takes into account target direction, obstacle avoidance, and distance. Prioritized replay concentrates training on important events while a dueling architecture separates value and advantage learning. The results show that the PMR-Dueling DQN has greatly increased convergence speed, stability, and overall performance across conditions. In both grid world and Gazebo environments the PMR-Dueling DQN achieved higher cumulative rewards. The combination of deep reinforcement learning with reward design, network architecture, and experience replay enables the PMR-Dueling DQN to surpass traditional approaches for robot path planning in complex environments.

## 1. Introduction

In recent decades, mobile robots have been utilized across various domains, such as the military, industry, and security, to perform vital unmanned tasks. A key challenge in enabling effective navigation for mobile robots in complex environments is path planning [1]. The principal goal of path planning is to identify the optimal trajectory between a robot’s initial and desired end positions. While there are typically multiple feasible paths to choose from, the best path is selected based on criteria such as minimum distance, path quality, energy efficiency, or, most commonly, the shortest distance and time [2]. The development of high-performance path-planning techniques has been crucial to allowing mobile robots to determine optimal routes amidst obstacles and avoid unnecessary detours.

In the area of mobile robot path planning, effective algorithms play a crucial role by considering factors such as path length and traversal time to optimize point-to-point routes. Recent years have witnessed a notable transformation driven by the application of deep reinforcement learning (DRL) algorithms and the widespread adoption of artificial intelligence in this domain. This shift has given rise to intelligent, flexible, and dynamic path-planning processes for mobile robots. The incorporation of AI and DRL has introduced several advantages, including the automation of path planning, the establishment of interconnected cyber-physical systems, and the continuous exchange of real-time data facilitated by cloud computing. This integration has led to the development of machine learning-based control algorithms, elevating the capabilities of mobile robot path planning in diverse applications such as manufacturing processes [3].

Path planning is generally divided into two categories: local path planning and global path planning [4]. Global path planning requires full knowledge of the environment in advance in order to pre-compute a collision-free path connecting the origin and destination prior to execution. Complete environmental information is needed upfront. Consequently, the main objective of global path planning is to create the environment and subsequently optimize the path [5]. In contrast, local path planning can handle unknown or partially known environments. Also called reactive methods, local techniques are used for navigating unfamiliar spaces where there is significant uncertainty. Local path planning requires that the robots gather data and information from their surroundings throughout the whole process, which requires the robots to be prepared with learning abilities. Local planners dynamically react in real time based on sensor feedback rather than relying on pre-mapped routes. In practical applications, it is common to employ a hybrid approach that combines both local and global planning methods because ensuring the reliability of global path planning becomes challenging in dynamic and complex environments when these approaches are employed. Global planning is typically updated at a lower frequency, considering the broader environment, while local planning is updated more frequently, responding to each new action the agent takes. A well-known example is the Move Base function package in the Robot Operating System (ROS). This hybrid strategy allows for efficient and adaptable navigation, leveraging the advantages of both global and local planning techniques.

In recent years, rapid advances in reinforcement learning have created new potential for addressing the long-standing challenge of path planning for mobile robots [6]. As both reinforcement learning theory and practical algorithms continue to evolve at a rapid pace, applying modern reinforcement learning techniques to robot path planning problems has become an active and promising research area for tackling these challenges. This is because reinforcement learning does not require prior knowledge of complex environmental models, instead enabling agents to learn competent behavior through direct interaction with their surroundings and feedback in the form of reward signals. This makes reinforcement learning a highly appealing approach for robot path planning applications. Reinforcement learning has been successfully utilized across a diverse array of domains, including autonomous vehicle control [7], advanced robotics [8,9], and, critically, mobile robot path planning [10]. In reinforcement learning, agents learn optimal behavior purely from experience by interacting with their environment and observing the associated perspectives and reward outcomes, eventually converging on learned value functions that allow maximally rewarding performance [11]. A key advantage is that reinforcement learning agents can acquire skills in rarely visited sections of state space that may lack human-provided examples [12]. However, while traditional Q-learning algorithms have shown promise for path planning, their slow convergence hinders effectiveness in addressing large-scale, complex real-world problems [13]. To tackle this issue, in this work, we used the Dueling Deep Q-Network introduced by [14] with the shaped Rewards and Prioritized Experience Replay (PMR-Dueling DQN) approach. By incorporating a shaped Reward function that takes into consideration target proximity, trajectory smoothing, and obstacle avoidance at each step, this method goes beyond traditional feedback signals. The algorithm’s performance in difficult navigation settings is greatly enhanced by the refined training signal that is the consequence of rigorous design and adaptation of each of the environmental aspects. We conducted a comparative analysis against the standard Q-learning, DQN, and DDQN approaches for global path planning in the grid map environment, as well as for both global and local path planning in the Gazebo simulation environment. Our evaluations across the grid world and Gazebo simulation environments with mobile agents show that the PMR-Dueling DQN algorithm outperforms the others in terms of average rewards accumulated, steps taken, and path lengths traversed.

In general, this paper has the following key contributions:

1. The PMR-Dueling DQN method for mobile robot path planning in complex and dynamic situations is used to address the problems of instability and slow convergence seen in the DQN algorithm. To achieve superior performance in terms of convergence speed, stability, and path planning performance, the algorithm integrates deep neural networks, dueling architecture, Prioritized Experience Replay, and shaped Rewards.
2. A more realistic Gazebo simulation setting is used in addition to the traditional grid map. The physical simulation platform, called Gazebo, bridges the gap between the real and virtual environments by offering a simplified model of the environment that is quite similar to the real one. This method guarantees the agent’s learned methods apply to actual robotic environments.
3. To address the influence of obstacles, a shaped Reward function is built for each environment model, contributing to the enhancement of the planning path and a boost in the convergence speed of the algorithm by incorporating obstacle avoidance and the distance metrics to the target position. This improvement aims to strike a balance between steering clear of obstacles and progressing toward the target, enabling the robot to devise a path that avoids the area of obstacles which addresses challenges associated with sparse reward problems.
4. A comparative analysis is conducted, wherein we evaluate the efficiency of our proposed method by comparing it with traditional Q-learning and other deep reinforcement learning (DRL) algorithms.

The remaining parts of this paper are organized as follows. In Section 2, Section 3, and Section 4, we introduce related work, the theoretical foundation, and the methodology, respectively. We conduct simulation experiments and analyze the results in Section 5. In the final section, Section 6, we summarize the work of this paper.

## 2. Related Work

Over the years, various conventional global planning techniques have been developed. Dijkstra’s approach [15] selects the node closest to the start and yields the shortest path but has a high computational cost compared to BFS and A* [16]. The probabilistic roadmap (PRM) method [17] utilizes graph search to discretize continuous space for algorithms such as A* search. The PRM is widely used for its ability to find short paths efficiently. The rapidly exploring random trees (RRT) approach [18] can construct real-time paths and handle high-dimensional spaces well but may achieve inferior path optimality versus Dijkstra or the PRM.

Reinforcement learning has made major advances in robot planning recently. Ref.  [19] developed multiple Q-networks for grid-based tasks, while [20] improved Q-learning convergence for grid path planning. However, Q-learning has limitations as the environment grows, needing prolonged computation for Q-value updates and larger memory matrices. Learned matrices may also be suboptimal. To address this, Ref. [21] integrated a metaheuristic into Q-learning to reduce optimal solution search times. Ref. [22] proposed a heuristic RL method that selects strategies by incorporating cost functions and heuristics. Adding cost learning balances rewards and costs in RL, validated on robot path planning problems. Backward Q-learning [23] combines Q-learning and Sarsa for faster convergence in various systems. Ref. [24] combined Q-learning and neural networks to efficiently handle dynamic and static obstacles for robot navigation. By presetting speeds, their technique simplifies time-critical applications. Evaluations showed positive results. Ref. [25] integrated Q-learning and differential evolution in a memetic algorithm, enhancing multi-robot navigation performance versus GA, PSO, and differential evolution.

Recently, the use of deep reinforcement learning (DRL) algorithms as the fundamental control [26], navigation [27], localization [28], and planning [29] systems has gained popularity. The flexibility of artificial neural networks, along with the capacity to simulate, train, and utilize them as end-to-end solutions, has garnered interest from the research community. This has motivated efforts to integrate these advancements from DRL into robotics applications. The adaptability of neural networks paired with the ability to train them through simulation makes them well suited for serving as comprehensive systems for various robot functions. RL decision making and neural network fitting are combined for planning in specific environments [30]. However, these methods have limitations such as dimensionality issues in complex spaces, slow convergence, and lack of portability [31]. To address these issues, Ref. [32] introduced the Improved Dueling Deep Double Q-Network (ID3QN) algorithm tailored for nuclear environments. This algorithm, leveraging an asymmetric neural network structure and a priority experience replay mechanism, addressed overestimation issues prevalent in traditional DQN algorithms. However, while excelling in radioactive grid scenarios, it might lack adaptability across varied complex environments and might not prioritize real-time obstacle avoidance. In [33], a novel algorithm integrating reinforcement learning with the Deep Q-Network (DQN) aimed to empower agents for real-time decision making and trajectory planning in Gazebo simulations. However, this approach, while promoting exploration, might overlook real-time obstacle avoidance during trajectory planning, potentially limiting its effectiveness in dynamic environments.

To address the overestimation issue in DQN, various improved algorithms have been proposed, such as Double DQN (DDQN) by [34], which uses two networks to estimate the value of actions to reduce overestimation. Exploratory Noise DQN (EN-DQN) by [35] adds noise during action selection and uses recurrent neural networks to capture multi-step changes affecting decision making. The competitive network method by [36] balances Q-value updates in the online and target networks to mitigate reward bias. The dynamic fusion DDQN by [37] incorporates prior knowledge into network training and adjusts weights to reduce overestimation. While these enhancements have improved DQN performance to some degree, issues remain with slow convergence and ineffective training. Employing only camera images, a method proposed by [38] used a Double Deep Q-Network (DDQN) algorithm with discrete actions to navigate an environment and successfully avoid obstacles. Although this method works well in some situations, its use of discrete actions affects performance in dense or complex environments, and its reward function avoids challenges with local minima. Similarly, Ref. [39] used a DDQN algorithm with discrete actions for navigation, rewarding objective achievement and collision avoidance based on distance. However, this approach requires the grid map occupancy as input, increasing runtime computational expenses. In contrast, our proposed approach prioritizes the design of a structured reward function that encompasses domain-specific objectives, including goal attainment, path smoothing, and obstacle avoidance. This is achieved while employing a comprehensive strategy that combines dueling architecture and Prioritized Experience Replay with shaped Rewards.

The PMR-Dueling DQN method directly addresses the sample efficiency limitation. Prioritized Experience Replay then prioritizes experiences based on their importance, which makes sure that rare and crucial experiences are used efficiently during learning. The combination also addresses the limitation of adaptability. Dueling DQN allows the agent to quickly adapt its decision-making process to changes in the environment by focusing on learning state and action values independently. We utilize neural networks instead of Q-tables and experience replay to improve learning while reducing data correlations. Extensive simulations demonstrate the technique is effective in efficient path planning. The PMR-Dueling DQN appears to effectively address robot path planning problems. This algorithm showcases robust performance across diverse scenarios. We purposely design a shaped Reward system that is in line with planning goals, compared to a naive signal. By incorporating smoothness, obstacles, and progress-rich elements inside the optimum reward, policies are guided to overcome persistent poor local convergences in starting point schemes. Basically, the carefully shaped reinforcement signal operates as the crucial driving force for quickly learning safe, effective navigation methods. Our method addresses statistical inefficiencies that limit deep reinforcement learning for robot planning by developing rewards specifically designed for navigation dynamics tasks. With its shaped Rewards prioritizing obstacle avoidance and navigation, could offer improved real-time decision-making capabilities and mitigate overestimation issues.

## 3. Theoretical Foundations

In this section, we provide an overview of the fundamental theories behind Dueling Deep Q-Network and Prioritized Experience Replay in which our proposed method builds upon these theories to improve the performance of traditional DQN.

**Figure 1:**
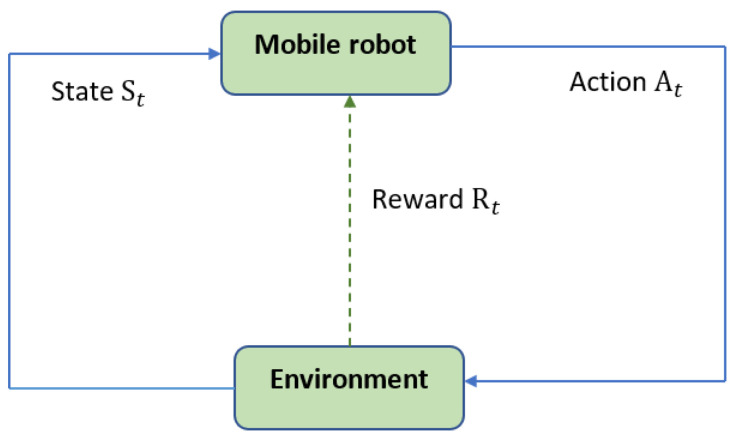
The reinforcement learning process.

Reinforcement learning involves an agent interacting with an environment, as depicted in Figure 1. It consists of a cyclic process of the agent observing the environment state, taking an action, receiving a reward, and updating its strategy. A common reinforcement learning technique is Q-learning, which iteratively improves the quality value (*q*) function that guides the agent’s action selection policy. This Q-learning update process follows a specific mathematical formula shown in Equation (1) for refining the *q*-values based on the state, action, reward, and next state observed during agent-environment interaction.

(1) $$q\left(s_{t},a_{t}\right)\leftarrow q\left(s_{t},a_{t}\right)+\eta \left(r_{t}+\gamma \max_{a^{\prime }}q^{*}\left(s_{t+1},a^{\prime }\right)-q\left(s_{t},a_{t}\right)\right)$$

where $s_{t}$ denotes the current state, $a_{t}$ the action taken, η a scaling coefficient, $r_{t}$ the observed reward, $s_{t+1}$ the next state reached, $a^{\prime }$ the possible next actions, and γ the discount factor on future rewards. However, the optimal $q^{*}$ values are initially unknown, so the algorithm relies on the current $q_{t}$ estimates to recursively replace the optimal $q^{*}$ through a process called bootstrapping. In essence, $q_{t}$ uses its own value to update itself to an improved $q_{t+1}$ at each timestep, progressively converging closer to the true optimal *q*.

### 3.1. Deep Q-Network (DQN)

DQN improves reinforcement learning by utilizing deep neural networks to solve complicated problems with high-dimensional states. The deep neural network’s primary job is to estimate the action value function, allowing the calculation of Q-values for all available actions in each state. The approximation function, designated as Q(S,a;), considers the current state, represented as S, and an action from the action space, denoted as a. The network weights, indicated by w, are critical in determining the approximation’s accuracy. The DQN algorithm can find the action with the highest Q-value in a given state by employing the deep neural network. This procedure guarantees that the agent makes accurate decisions according to the estimated values of various actions. Iterative training updates the network’s weights, w, allowing for continuous improvement in Q-value approximation. As a result, the agent trains to maximize its behavior by selecting actions that correlate to the highest Q-value in the current state.

Generally, the DQN method applies deep learning to tackle reinforcement learning problems involving complex and continuous state and action spaces. It builds a value network and a loss function, with the loss function measuring the difference between the network’s anticipated and desired values [40]. The objective is to minimize this disparity by adjusting the network’s parameters. Training the Q-network requires the use of labeled samples. Equation (2) represents the modification of the Q-learning algorithm, which is the core of DQN.

(2) $$\begin{array}{cc} Q\left(S_{t},A_{t}\right) & \leftarrow a\left(S_{t},A_{t}\right)+\alpha \left[R_{t+1}+\gamma \max Q\left(S_{t+1},A_{t}\right)\right.\\ \; & \;\left.-Q\left(S_{t},A_{t}\right)\right] \end{array}$$

Then, the loss function can be defined as follows in Equation (3).

(3) $$L\left(\omega \right)=E\left[{\left(R_{t+1}+\gamma \max Q\left(S_{t+1},A_{t}\right)-Q\left(S_{t},A_{t},\;\omega \right)\right)}^{2}\right]$$

Throughout the network training process, training data are constantly gathered and stored as training labels in an experienced pool. The network is then trained on these data from the experience pool using small batches and random gradient descent methods. The objective is to optimize the weights of the network and solve the Q-value function.

### 3.2. Deep Dueling Neural Network (Dueling DQN)

Research indicates deep Q-learning can struggle with overestimated values, hindering convergence speed in problems with expansive action and state spaces [14]. Thus, we employ a deep dueling algorithm [14] introduced by Google DeepMind in 2016, combined with a shaped Reward function and Prioritized Experience Replay to further optimize convergence for this system. The key innovation making deep dueling superior is its neural network architecture. For many states, estimating action values is unnecessary when the choice of those actions does not impact what happens next [29]. The deep dueling architecture addresses this by separately learning state values and action advantages, rather than direct action values. This avoids wasted computation on inconsequential actions, accelerating training. Our use of deep dueling is motivated by its potential to mitigate overestimation and more quickly converge to optimal Q-values based on this improved neural network structure. Rather than directly estimating the action-value (Q) function, the deep dueling algorithm splits the neural network into two separate streams of fully connected layers. One stream estimates state value V(s;β), while the other estimates action advantages A(s,a;α), α, and β are the parameters of the two streams. These are combined at the output layer to produce the final Q-values as shown in Equation (4).

(4) Q(s,a;α,β)=V(s;β)+A(s,a;α)

By separating value and advantage estimations, deep dueling avoids wasted computation on action values that do not influence subsequent states. However, the separate state value *V* and action advantage *A* are not identifiable because the network directly outputs Q-values. To address this, the advantage function *A* is centralized by subtracting its average over all actions $a^{\prime }$. This centers the advantages while ensuring performance and improving optimization stability. Here, $meanA(s,a^{\prime };\alpha )$ represents the mean advantage over all actions $a^{\prime }$ with parameters α. The resulting modified Q-values calculated from the centrally aligned advantages are shown in Equation (5). By centralizing the advantage, the deep dueling algorithm can achieve stable optimization despite the lack of identifiable separate value and advantage terms.

(5) $$\begin{array}{cc} Q(s,\;a\;;\;\alpha ,\;\beta ) & =V\left(s;\;\beta \right)+(A\left(s,\;a\;;\;\alpha \right)-meanA\left(s,\;a^{\prime }\;;\;\alpha \right)) \end{array}$$

### 3.3. Prioritized Experience Replay

The agent’s experiences including both successful and failed attempts are recorded during interaction with the environment and stored in the experience replay unit for training purposes. These events are recorded in the playback unit as they happen throughout the learning process. Replaying these incidents enables the agent to learn from both good and bad actions, enabling it to continuously improve its behavior. Using the recorded experiences, the agent gains an understanding of the effects of various actions and can make adjustments to enhance performance over time. Prioritized Experience Replay chooses events from the replay buffer based on their assigned priority rather than sampling events at random. Based on these preferences, it prioritizes experiences and probabilistically samples them. By emphasizing more important events, Prioritized Experience Replay has the potential to enhance learning effectiveness when compared to random sampling in experience replay.

However, the significance of various experience samples varies. Given that the experience replay unit is constantly updated with new samples, selecting a small number of samples using uniform random sampling as the model’s input could lead to inadequate utilization or even the overwriting of important data. The model’s training effectiveness suffers as a result. To increase the success rate of model training, this work uses the Prioritized Experience Replay technique [41]. With this tactic, choosing samples from the experience replay unit is prioritized, improving the likelihood of receiving samples with more significance. This increases the effectiveness of the training process, ensuring that meaningful experience samples contribute more significantly to the model’s learning and overall performance.

Each replayed event’s TD error is calculated again during the learning process, and the priority of the experiences is adjusted by the new TD error. As shown in Equation (6), it uses a proportional prioritization method to decide how important experience is. The small positive integer ϵ ensures that samples with a TD error close to 0 have a negligible chance of being chosen.

(6) $$P_{l}=\left|\delta_{1}\right|+\epsilon$$

As a result, Equation (7) can be used to calculate the likelihood of choosing experience *l*. In this equation, m represents the size of the experience replay unit. The parameter α, which falls within the range [0, 1], governs the degree of priority utilization. α=0, uniform sampling is employed, while α=1 corresponds to a greedy strategy sampling approach.

(7) $$P_{l}=\frac{p_{l}^{\alpha }}{\sum_{m}p_{m}^{\alpha }}$$

## 4. Methodology

**Figure 2:**
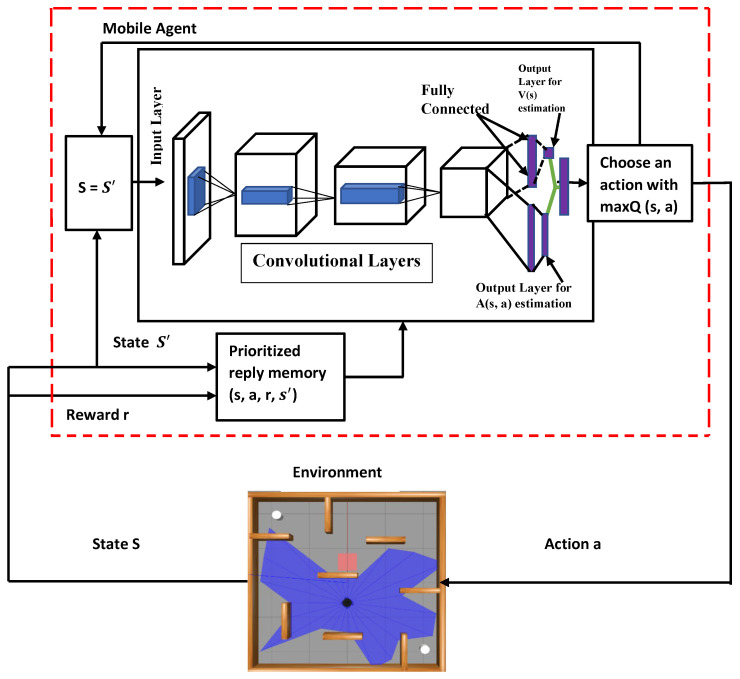
The proposed algorithm’s framework.

In this section, the framework of the proposed Dueling Deep Q-Network with shaped Rewards and Prioritized Experience Replay (PMR-Dueling DQN) is described in detail with the design of the respective reward function. The path planning system for the mobile robot depicted in Figure 2 is an enhanced Dueling DQN (PMR-Dueling DQN) system that prioritizes experience replay. To replace the traditional Q-table, we use a dueling network in our solution. To give the agent more information when choosing which actions to perform, the network learns two distinct representations of the state-action value function: the state value and the advantage. In comparison, the action advantage function correlates with the action and signifies the average perceived merit of the action concerning the state. It effectively addresses the reward-bias issue. With this competitive network structure, the agent can grasp a more authentic value V(s) reflective of the environmental state, devoid of any action-related influence [14]. While Dueling DQN provides an architectural modification to improve state value estimates, it does not fully address the underlying challenge of reward sparsity in complex tasks. In scenarios where local information is restricted, the presence of sparse and delayed rewards poses substantial challenges in the area of robotic navigation. For example, a reward structure that only acknowledges the agent upon goal attainment or penalizes collisions with obstacles can lead to various issues. In certain situations, a trained policy might exhibit movements toward the goal and actions in the opposite direction to navigate around obstacles, effectively trapping the agent at a local minimum within the environment. Additionally, acquiring effective escape strategies may necessitate exposure to a diverse set of scenarios.

To address this, a shaped Reward function *r* is introduced in Section 4.4 to mitigate the limitations inherent in Dueling DQN’s improvements by offering a more structured and guided learning process. Specifically, instead of only giving a large reward for completing the entire task, intermediate rewards are introduced to reinforce incremental progress. This encourages the agent to adopt intermediate strategies, minimizing the risk of being stuck at a local minimum. By incorporating this shaped Reward function, the model receives targeted feedback, influencing the learning dynamics to emphasize crucial aspects that the network might overlook. Q(S, a) indicates the approximation function used, where S represents the current state and a represents the action inside the action space. As a result, in a given state, the action having the highest possible Q value is chosen as the optimal choice for the robot.

### 4.1. Simulation Environments and Setup

In order to verify the effectiveness of the proposed algorithm, we conducted a comparative experiment in three training environments, which are a virtual 25 by 25 grid obstacle environment and two Gazebo simulation environments named E-1, E-2, and E-3, respectively. The two Gazebo simulation environments that resemble a real-world environment are: (1) an environment with static obstacles and (2) an environment that contains both static and dynamic obstacles. We used the Turtlebot3 robot featured by Gazebo as a platform for testing in simulation environments.

**Figure 3:**
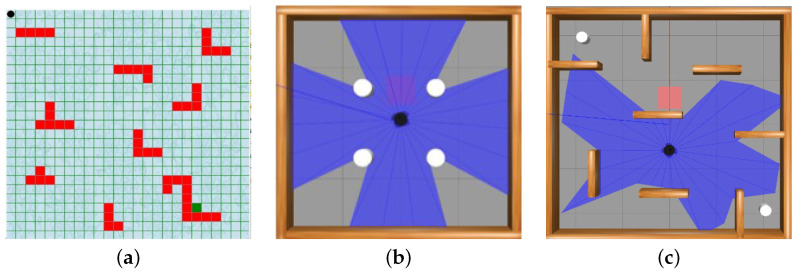
Simulation Environments: (**a**) a 25 × 25 grid map; (**b**) Gazebo environment with static obstacles; and (**c**) Gazebo environment that contains both static and dynamic obstacles.

A Python built-in standard library called Tkinter was used to build a GUI environmental model E-1. There is a 25 by 25 grid obstacle environment for this experiment. The white line around the outside of Figure 3a represents the boundary, while the green line within the whole map represents the grid. The inside gray surface depicts areas of the map that are free of obstacles, whereas the red surface symbolizes difficulties. The beginning position in the obstacle environment is in the upper left corner (1,1), while the target point is in green, towards the bottom right corner (19,21). The obstacle environment developed consists of 51 static obstacles of the same size that are positioned on the map by the coordinates of two diagonal points according to the Tkinter toolkit. The agent is represented by the black circle drawn inside the agent’s location, and the mapping environment in the code can be customized by modifying the grid coordinates.

As shown in Figure 3b,c, environment E-2 features four stationary cylindrical obstacles, while environment E-3 presents a more complicated setting with brown objects serving as static obstacles and two white cylinders positioned at the environment’s corners, serving as mobile obstacles. In both E-2 and E-3, a Turtlebot3 equipped with a laser range sensor operates as the robotic platform. A red rectangle object denotes the target. Throughout the training phase, the robot’s initial pose is set as the geometric center of the training environment’s floor. The target’s placement occurs randomly in positions devoid of obstacles within the environment. This experimental setup facilitates the investigation of the robot’s ability to navigate through diverse environments, dealing with static and dynamic obstacles. The selection of the Turtlebot3 platform and the specific range of the laser sensor contribute to a comprehensive evaluation of the proposed algorithm in varying scenarios, thereby enhancing the robustness and effectiveness of the algorithm’s path-planning capabilities. The total number of episodes in the simulation experiment is set to 3000 in the grid map environment E-1 and 1000 in the two Gazebo simulation environments E-2 and E-3. An episode is terminated after the agent either reaches the goal or collides with an obstacle. The training concludes when the agent’s average reward reaches a satisfactory and consistent level. The model used in this work was trained using the software and hardware specifications outlined in Table 1. The algorithm was implemented in Python 3.7 using TensorFlow as the backend for Keras to define the neural network architecture. Leveraging TensorFlow allowed the model to take advantage of GPU acceleration during training.

### 4.2. State of the Environment

In the simulated environments utilized for this study, the design of the state aimed to summarize crucial information vital for effective navigation and obstacle avoidance.

The state representation in the Tkinter-based grid environment (E-1) consisted of the agent’s exact location inside the grid, the Euclidean distance to the target, and indicators denoting the proximity of obstacles to the agent’s position. So, the state vector $s_{t}$ of environment E-1 at time step t can be represented as follows:

(8) $$S_{t}=\left(x_{agent},\;y_{agent},\;d_{target},\;O_{proximity}\right)$$

where $x_{agent}$ and $y_{agent}$ denote the Cartesian coordinates of the agent’s position within the grid. $O_{proximity}$ is a binary indicator vector signifying the presence or absence of obstacles within the agent’s vicinity. $d_{\mathrm{target}}$—the Euclidean distance between the agent and the target point—would be:

(9) $$d_{\mathrm{target}}=\sqrt{{\left(x_{\mathrm{agent}}-x_{\mathrm{target}}\right)}^{2}+{\left(y_{\mathrm{agent}}-y_{\mathrm{target}}\right)}^{2}}$$

where $x_{\mathrm{agent}}$, $y_{\mathrm{agent}}$ denote the Cartesian coordinates of the agent and $x_{\mathrm{target}}$, $y_{\mathrm{target}}$ denote the Cartesian coordinates of the target.

**Figure 4:**
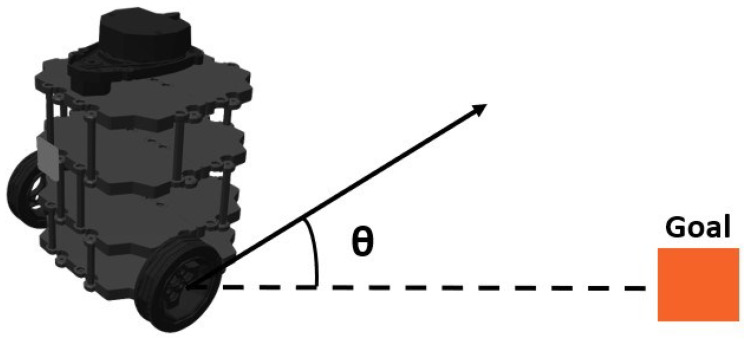
Robot orientation with current position and goal heading: sensor-based navigation.

In contrast, the Gazebo simulation environments (E-2 and E-3) included a more dynamic state representation that contained the Turtlebot3 robot’s pose, information from the laser range sensor about the distances of obstacles in different directions, the coordinates and properties of both stationary and moving obstacles and the target position’s distance from the obstacle. Seven Lidar points are properly configured in the Gazebo simulation. The given value strikes a balance between the inputs required by the control system and the use of computational resources. Through careful selection of the Lidar points, the control system can maintain computational efficiency while obtaining pertinent environmental data. With this complete understanding of its surroundings, the control system may make use of the state information to assess the environment, identify any obstacles, and map out the best routes around them. The Lidar sensor’s precise and comprehensive perception significantly enhances the robot’s overall performance and makes it possible for it to safely and effectively navigate challenging environments. These state variables were carefully chosen to provide relevant environmental signals to the reinforcement learning agent so that it could make decisions and avoid obstacles in the simulated environments. The laser scanner covers a detection range of angle θ from 0 to 360 degrees, thereby creating a 360-dimensional data representation as shown in Figure 4. To address the challenge of dealing with high dimensionality while ensuring the capture of environmental details, we constrained the detection distance to fall within the range from 0.12 m to 3.5 m.

(10) $${\mathrm{pose}}_{robot}=\left(x,y,\theta \right)$$

where *x* is the x-coordinate of the Turtlebot3 robot; *y* is the y-coordinate of the Turtlebot3 robot; and θ is the angle between the robot’s current position and direction to the goal. So, for environments (E-2 and E-3) with the Turtlebot3 equipped with a laser range sensor, the state $s_{t}$ can be represented as follows.

(11) $$S_{t}=\left({\mathrm{pose}}_{\mathrm{robot}}+\;{\mathrm{sensor}}_{\mathrm{laser}}\left(7points\right)+\;{\mathrm{obstacle}}_{\mathrm{info}}+\;d_{\mathrm{target}}\right)$$

where ${\mathrm{sensor}}_{\mathrm{laser}}$ contains data from the laser range sensor, providing distance measurements from the seven Lidar points to surrounding obstacles in multiple directions. ${\mathrm{obstacle}}_{\mathrm{info}}$ includes details about the static and mobile obstacles such as their positions, shapes, and properties. $d_{\mathrm{target}}$ signifies the distance from the robot’s current location to the specified target point.

### 4.3. Network Structure

The Tensorflow library was used due to its open-source nature and ability to offer versatile machine-learning tools. Its API enables the building of models and applications with an adjustable architecture. Keras was used to program the network layers, reducing the amount of code needed to construct control systems.

**Figure 5:**
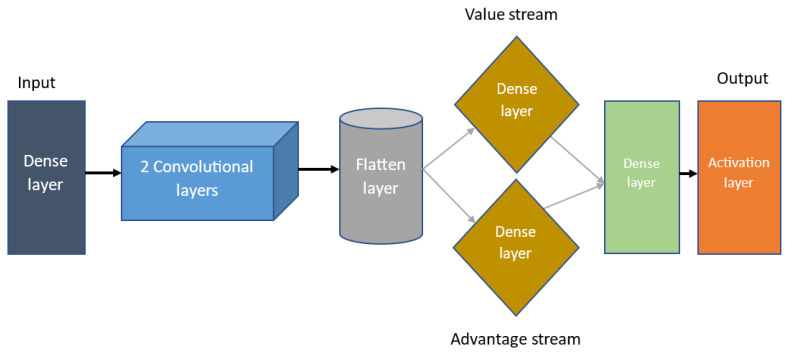
Architecture for the PMR-Dueling DQN network.

The network model architecture shown in Figure 5 is planned in a step-by-step fashion. The network consists of an input layer implementing a dense ReLU activation to accept the state representation input data. This feeds into two convolutional layers which extract spatial features from the input states. The output is then flattened into a 1D vector and split into separate advantage and value streams. Both the advantage stream and the value stream contain dense layers with ReLU activation to estimate the advantage function and value function, respectively. After the final advantage and value functions are combined by the aggregation layer, it is then passed to a linear activated output layer representing the Q-value for each possible action in the given input state. Using a linear output activation allows the network to predict Q-values unconstrained to any range.

The network model has an input layer that contains key state information to inform the action selections of the output layer. The dimensions of these input and output layers differ slightly based on whether the agent is operating in the Gazebo simulation environment or the abstract grid world map.

In the case of the Gazebo 3D simulator, the state representation fed into the input layer consists of 10 variables describing aspects such as the agent’s current position, orientation, recent velocity, goal location, and obstacle proximity. Therefore, the input layer contains 64 neurons to match this 10-dimensional state space representation.

The output layer for the Gazebo environment consists of 7 neurons, each encoding one of the allowable actions. There are 2 actions for linear velocity control (forward/back) and 5 actions for angular velocity control (turning left/right and three different turn speeds). When the agent is alternatively placed in the 2D grid world map, the state representation contains only 4 dimensions—the x-y coordinates of the agent and goal. Correspondingly, the input layer has 64 neurons to match this reduced 4-dimensional state space. The grid world affords just 4 movement actions—up, down, left, right. So, the output layer consists of 4 neurons, each encoding one of these discrete navigation directions.

### 4.4. Design of Shaped Reward Function

In a reinforcement learning context, the agent’s behavior is greatly influenced by the reward function. The proposed reward function summarizes a unique balance of alignment, distance, and obstacle avoidance rewards tailored explicitly for mobile robot navigation tasks. Unlike singular reward structures, which often focus on singular aspects, our approach merges these components to holistically address challenges in path planning. This distinctive combination aims to incentivize the agent’s behavior by not only encouraging progress toward the goal but also ensuring optimal orientation alignment, efficient path selection, and proficient obstacle avoidance, thereby contributing to a more comprehensive learning framework for mobile robots. In this paper, we propose a reward function for the grid map environment (E-1), named $r_{{env}_{1}}$, and the Gazebo environments (E-2 and E-3), named $r_{{env}_{2,3}}$, respectively.

A small reward at each time step, a penalty for crashing into an obstacle, or a sparse reward for completing numerous steps to reach the goal without colliding may be sufficient to train an agent trying to explore its environment while avoiding obstacles to the task at hand. So, for environment E-1, we calculate the reward (cost) for 3000 episodes, and the reward function is defined in Equation (12):

(12) $$r_{{\mathrm{env}}_{1}}=\left\{\begin{array}{cc} 10, & \mathrm{if}\;\mathrm{the}\;\mathrm{agent}\;\mathrm{reaches}\;\mathrm{the}\;\mathrm{target}\\ 0, & \mathrm{if}\;\mathrm{the}\;\mathrm{agent}\;\mathrm{reaches}\;\mathrm{any}\;\mathrm{position}\\ -1, & \mathrm{if}\;\mathrm{the}\;\mathrm{agent}\;\mathrm{reaches}\;\mathrm{an}\;\mathrm{obstacle} \end{array}\right.$$

The reward function of $r_{{env}_{2,3}}$ is comprised three components; the alignment reward $r_{a}$, distance reward $r_{d}$, and obstacle avoidance reward $r_{ob}$. We proposed the reward function to align with the operational characteristics of the robot, taking into account the specific values associated with the robot used in our simulations for training Turtlebot3. These values included a linear velocity of 0.2 m/s or 0.4 m/s and angular velocities of $\pm \frac{\pi }{6}\;\mathrm{rad}/s,\pm \frac{\pi }{12}\;\mathrm{rad}/s,0\;\mathrm{rad}/s$. The robot’s progress towards the target is directly influenced by its linear velocity. We made the distance reward ($r_{d}$) component more sensitive to the linear velocity variations to account for this. Then, we extended the alignment reward ($r_{a}$) component to account for angular velocity variances, taking into consideration the range of angular velocities the robot can handle. The reward is now influenced by the difference between the current heading direction and the angle to the goal, considering the specific angular velocity values. It is calculated for 1000 episodes. The agent is encouraged to align its orientation with the objective direction through $r_{a}$. It is calculated based on the discrepancy between the agent’s heading and the angle towards the goal. As the angle deviates from the ideal alignment, the reward falls and is highest when the agent is traveling straight for the target.

(13) $$r_{a}=1-4\left|0.5-\left(0.25+0.5\times \frac{\left(\theta \%(2\pi )\right)}{\pi }\right)\right|$$

where θ is the difference between the current heading direction and the angle to the goal. So, θ%(2π) would yield the remainder when the value of θ is divided by 2π. The result will be a value between 0 and 2π.

An exponential function is used to calculate the distance reward $r_{d}$.

(14) $$r_{d}=2^{\left(\frac{d_{c}}{d_{g}}\right)}$$

where $d_{c}$ is the current distance between the agent and the goal and $d_{g}$ is the desired or optimal distance between the agent and the goal.

The obstacle avoidance penalty $r_{ob}$ is a set negative reward for approaching obstacles too closely:

(15) $$r_{ob}=\left\{\begin{array}{cc} -5, & \mathrm{if}\;min\_range<0.5\\ 0, & otherwise \end{array}\right.$$

where min_range is the minimum distance from the agent to the nearest obstacle detected by its sensor. It is calculated based on the data from the laser range finder in the environment. Combining Equations (13)–(15), the total reward function can be calculated in Equation (16) as follows.

(16) $$r=\left(round\left(r_{a}\left[\mathrm{action}\right]\times 5\right)\times 2^{\left(\frac{d_{c}}{d_{g}}\right)}\right)+r_{ob}$$

where the round function is applied to ensure that the computed reward is represented as a discrete value, enhancing the interpretability of the reward signal.

Therefore, the whole reward function for the agent in the Gazebo simulation environments is expressed in Equation (17).

(17) $$r_{{env}_{2,3}}=\left\{\begin{array}{cc} 500, & d_{c}=d_{g}\\ r+r_{ob}, & d_{c}<d_{g}<d_{ob}\\ -100, & d_{o}b<d_{g} \end{array}\right.$$

where $d_{ob}$ is the distance between the current distance and the obstacle.

So, the total reward functions for the grid map and Gazebo simulation environments; $r_{{env}_{1}}$ and $r_{{env}_{2,3}}$, respectively, are defined as follows in Equation (18).

(18) $$r_{t}=\left\{\begin{array}{c} r_{{\mathrm{env}}_{1}}=\left\{\begin{array}{cc} 10, & \mathrm{agent}\;\mathrm{reaches}\;\mathrm{the}\;\mathrm{target}\\ 0, & \mathrm{agent}\;\mathrm{reaches}\;\mathrm{any}\;\mathrm{position}\\ -1, & \mathrm{agent}\;\mathrm{reaches}\;\mathrm{an}\;\mathrm{obstacle} \end{array}\right.\\ r_{{env}_{2,3}}=\left\{\begin{array}{cc} 500, & d_{c}=d_{g}\\ r+r_{ob}, & d_{c}<d_{g}<d_{ob}\\ -100, & d_{o}b<d_{g} \end{array}\right. \end{array}\right.$$

## 5. Performance Evaluation

In this section, the parameter settings, simulation results, and performance comparison analysis of the proposed PMR-Dueling DQN method, shown in Algorithm 1, are presented. The method is compared with the classical Q-Learning, DQN, and DDQN algorithms.

### 5.1. Parameter Settings

After several experiments, the optimum parameters are suggested in Table 2 which are the modest values for learning, indicating that the algorithm will indeed maintain old information whilst still enabling new data to be obtained. α and β are the parameters to control the amount of prioritization and importance sampling, respectively.
**Algorithm 1** Dueling Deep Q-Network with shaped Rewards and Prioritized Experience Replay (PMR-Dueling DQN).1:Input: minibatch *m*, replay period *K*, action advantage parameter α, state value parameter β, budget *T*2:Initialize replay memory *D*, weight-change Δ3:Initialize Dueling Q-Network parameters θ4:Initialize the target Dueling Q-Network parameters $\hat{\theta }=\theta$5:Initialize $r_{t}$ of Equation (18)6:Observe initial state $S_{0}$ and select initial action $A_{0}$ using the online network $\pi_{0}\left(A_{0}|S_{0}\right)$7:**for**t=1 to *T* **do**8:    Observe state $s_{t}$, reward $r_{t}$, next state $s_{t+1}$, and random action $a_{t}$9:    Store transition $\left(s_{t},a_{t},r_{t},s_{t+1}\right)$ in the replay memory *D* with an initial priority value.10:    Combine the advantage and value functions as follows:
$$Q\left(s,a;\alpha ,\beta \right)=V\left(s;\beta \right)+(A\left(s,a;\alpha \right)-\mathrm{mean}A\left(s,a^{\prime },\alpha \right))$$11:    **if** tmodK=0 **then**12:        **for** j=1 to *m* **do**13:           Sample a transition $\left(s_{j},a_{j},r_{j},s_{j+1}\right)$ based on the priorities $P\left(j\right)\propto \frac{{P_{j}}^{\alpha }}{\sum_{j}{P_{j}}^{\alpha }}$14:           Update the priority of the transition to $|\delta_{j}|$15:        **end for**16:    **end if**17:    Update the network $\hat{\theta }=\theta$18:**end for**

### 5.2. Simulation Results and Performance Analysis

#### 5.2.1. For the Grid Map Environment (E-1)

**Figure 6:**
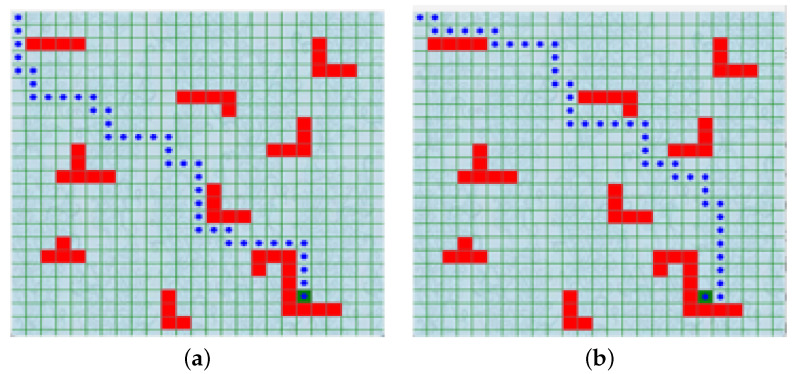

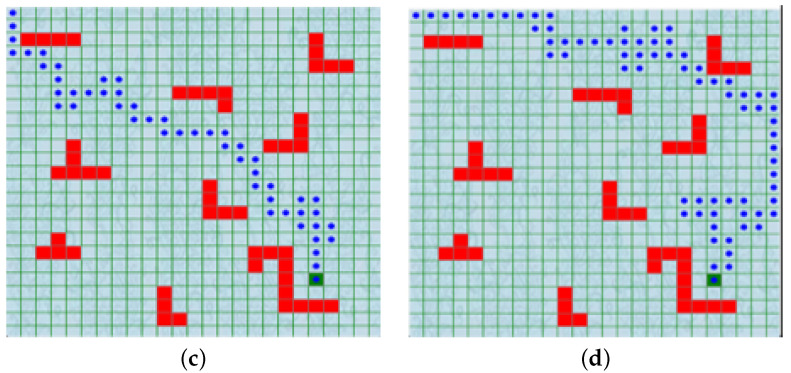
The shortest route drawn by the agent: (**a**) for the PMR-Dueling DQN algorithm; (**b**) for the DDQN algorithm; (**c**) for the DQN algorithm; and (**d**) for the Q-learning algorithm.

The mobile agent was given the responsibility of averting collisions with obstacles. Throughout the whole process, the agent could perform one of four actions: move up, move down, move right, or move left. The border and angle positions are exceptions, as one or more action options are not available. In addition, the agent could only take a step in each of the selected routes in a single action. The proposed environment is divided into cells, and some of those spaces are filled by barriers. If the mobile agent crashes into one of them, it is considered a collision (negative reward). Figure 6 shows the path gained after the model was trained for the proposed PMR-Dueling DQN algorithm, DDQN algorithm, DQN algorithm, and Q-learning algorithm, respectively. As is shown, the path planning result of the PMR-Dueling DQN algorithm is better than all of the other three algorithms while DDQN performs better than DQN. DQN also performs better than the classical Q-learning algorithm.

Table 3 shows the final Q-table with their respective optimal actions from the final route for the PMR-Dueling DQN algorithm. The training process in each run generates a Q Table, which represents the desired action for each location. The four actions shown in the table are according to the following sequences: action to the up, action to the down, action to the right, and action to the left. The Q-values of the completed table indicate the optimal actions the agent decided after observing the environment. Some of the sequences of final actions to achieve the goal after the agent’s full action are shown as follows: down-down-down-down-right-down-down-right-right-right-right-down-right-down-down-right-right-right-right-down-down... etc. As is evident, the Q-table stores the reinforcement learning agent’s learned knowledge about the optimal policy through its exploration of the environment. Analyzing the table provides insights into how the states are valued and which actions were preferred in certain states. As the agent explored the states and refined the Q-values through updating rules, useful state-to-action mappings were learned. Examining tables generated at different stages of learning can provide a better understanding of how those mappings evolved from mostly random to more optimal focused policies. The learned Q-table essentially represents the summarized experiences of the agent which determine its behavioral policy.

The technique for modifying the Q value is the most substantial difference between the four algorithms. The Dueling Deep Q-Network with Modified Rewards and Prioritized Experience Replay (PMR-Dueling DQN) algorithm gives more weight to experiences that provide valuable learning opportunities by combining deep neural networks, dueling architecture, shaped Rewards, and Prioritized Experience Replay, which can effectively learn control policies directly from raw sensory data in the given navigation environments resulting in improved training and potentially better action selection decisions compared to DDQN, DQN, and Q-learning. This provides much better data efficiency compared to tabular methods. The PMR-Dueling DQN also improves upon the original DQN algorithm by utilizing a dueling architecture to separately learn state value and action advantages. This helps mitigate overestimation bias, an issue that plagues both traditional Q-learning and DQN.

At the beginning of training, the visibility of agent movements from one place to another was intentionally slowed down to make it feasible to see and analyze its performance while adjusting the variables. A total of 35 experiments were conducted for all four algorithms with the same hyperparameters to obtain the optimal results.

**Figure 7:**
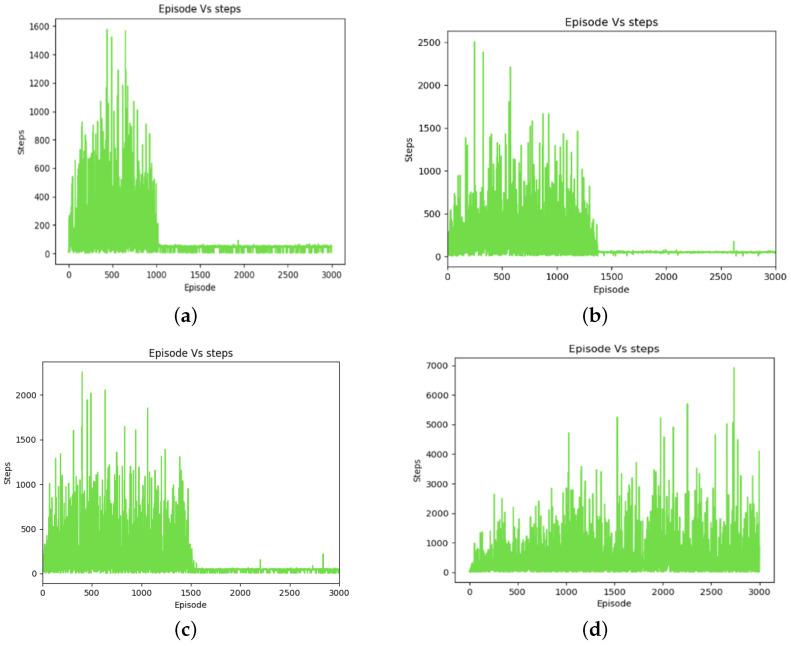
Training graphs of step changes over episodes: (**a**) for the PMR-Dueling DQN algorithm; (**b**) for the DDQN algorithm; (**c**) for the DQN algorithm; and (**d**) for the Q-learning algorithm.

As is shown in Figure 7, the PMR-Dueling DQN algorithm significantly outperformed the other methods in reaching the destination, converging in only 1000 training episodes. DDQN took 370 more episodes to locate the goal while basic DQN required 700 additional episodes. Tabular Q-learning exhibited no clear convergence trend even after being run for 3000 episodes, highlighting its poor sample efficiency. The PMR-Dueling DQN’s combination of deep neural networks, dueling architecture, Prioritized Experience Replay, and shaped Rewards enabled it to find optimal navigation policies with far greater speed and stability compared to DQN, DDQN, and tabular Q-learning.

**Figure 8:**
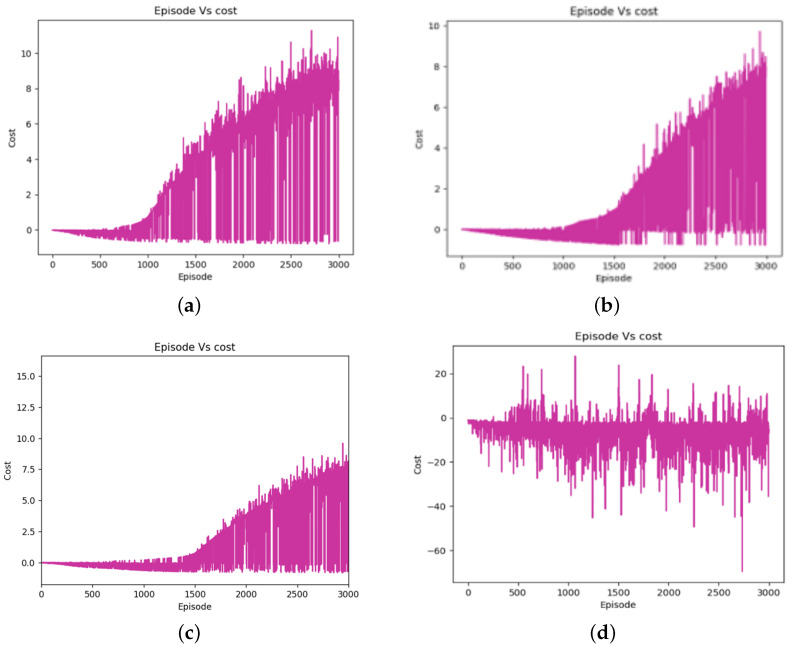
Training graphs of cost over episodes: (**a**) for the PMR-Dueling DQN algorithm; (**b**) for the DDQN algorithm; (**c**) for the DQN algorithm; and (**d**) for the Q-learning algorithm.

Figure 8 shows that the PMR-Dueling DQN algorithm clearly achieved the best performance in terms of maximizing cumulative reward, reaching a maximum cumulative reward of 10 over the training episodes. This high average reward demonstrates the PMR-Dueling DQN’s ability to quickly and stably converge to an optimal navigation policy. DDQN attained a decent but lower average cumulative reward of eight, taking more episodes to learn an effective policy. DQN performed even worse, only accumulating a maximum average reward of six due to its tendency to overestimate values during training. Meanwhile, traditional Q-learning exhibited extremely poor cumulative rewards, acquiring negative costs for nearly all training episodes and completely failing to find any positive reward policies. This comparison highlights the significant advantages of the PMR-Dueling DQN, which combines innovations such as shaped Rewards and Prioritized Experience Replay with deep neural networks to substantially outperform the DQN, DDQN, and Q-learning methods in both sample efficiency and policy optimality for robot navigation.

Table 4 summarizes the experiment’s outcomes in the grid map simulation environment. In terms of determining the ideal path, the simulation results show that PMR-Dueling DQN outperforms all the other three algorithms. From the results, it can be concluded that the Dueling Deep Q-Network with Modified Rewards and Prioritized Experience Replay (PMR-Dueling DQN) algorithm is better suited than the classical Q-learning, DQN, and DDQN algorithms for urgent path planning.

#### 5.2.2. For the Gazebo Simulation Environments (E-2 and E-3)

**Figure 9:**
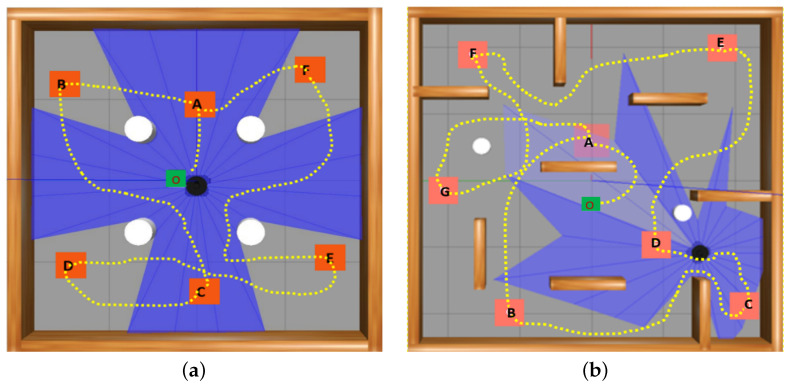
Generated path for the proposed PMR-Dueling DQN algorithm: (**a**) In environment E-2 which only contains static obstacles. The trajectory of the robot consisted of positions A to F, ultimately returning to the final destination at position A; (**b**) In environment E-3 which contains both static and dynamic obstacles. The robot followed a path that included positions A to G, and then returned to the final goal position A.

In the test environments E-2 and E-3, there were six and seven selected navigation points, respectively. The robot’s task was to independently explore these unfamiliar environments, commencing its movement from the initial point O. In the case of E-2, the robot’s path encompassed positions A through F, followed by a return to the final goal position A, all achieved without encountering collisions. Similarly, for E-3, the robot’s trajectory comprised positions A to G, followed by a return to the final goal position A, while ensuring collision-free movement, as illustrated in Figure 9. The local planner needs to be robust in handling both static and dynamic obstacles. It can utilize sensor data effectively to detect and react to dynamic obstacles. During planning, the global planner considers both static obstacles (from the map) and the current state of dynamic obstacles (from the Lidar sensor of the Turtlebot3 robot) to generate the target sequence. The local planner continuously monitors the environment E-3 for changes in dynamic obstacles and adapts its path accordingly which involves replanning the path if an obstacle suddenly appears on the planned trajectory. For example, in Figure 9b, a dynamic obstacle blocks the path between points F and G; the local planner detects this obstruction and replans a new collision-free path to reach the next goal point. The global path remains the same, but the local planner dynamically adjusts the specific motions to handle changes in the environment such as moving obstacles.

**Figure 10:**
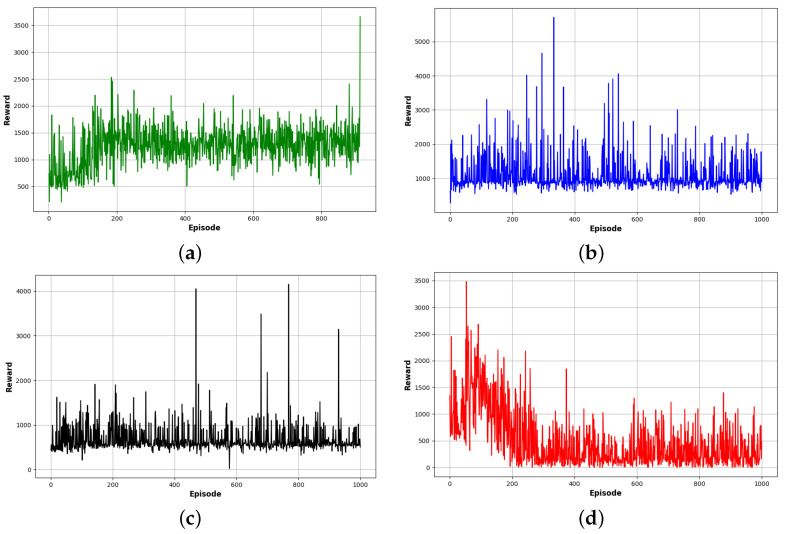
Cumulative rewards for each algorithm in environment E-2: (**a**) for the PMR-Dueling DQN algorithm; (**b**) for the DDQN algorithm; (**c**) for the DQN algorithm; and (**d**) for the Q-learning algorithm.

**Figure 11:**
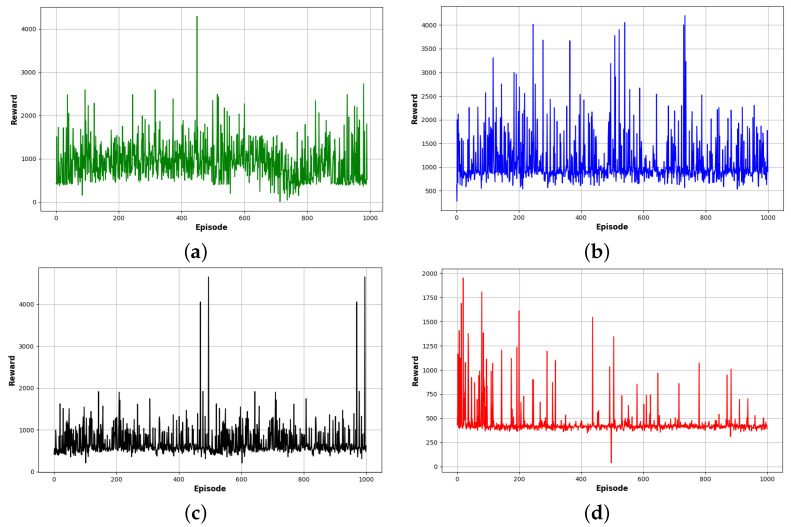
Cumulative rewards for each algorithm in environment E-3: (**a**) for the PMR-Dueling DQN algorithm; (**b**) for the DDQN algorithm; (**c**) for the DQN algorithm; and (**d**) for the Q-learning algorithm.

**Figure 12:**
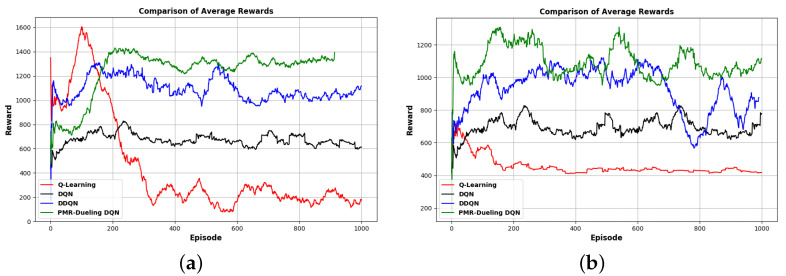
The robot’s reward comparison of the four algorithms: (**a**) in environment E-2, which only contains static obstacles, and (**b**) in environment E-3, which contains both static and dynamic obstacles.

In order to verify the efficiency of the proposed PMR-Dueling DQN algorithm, we conducted comparative experimentation in both Gazebo simulation environments. The cumulative rewards of each algorithm for the agent are shown in two separate figures, one for each environment. Figure 10 shows the cumulative rewards for each algorithm in environment E-2, and Figure 11 shows the cumulative rewards for each algorithm in environment E-3. Figure 12, compares the average rewards of the four algorithms for each environment. As shown in the figures, in the less complex environment E-2, the PMR-Dueling DQN algorithm again clearly outperforms the other algorithms, converging to a significantly higher reward of 1400 compared to 200 for Q-learning, 600 for DQN, and 1100 for DDQN. The performance gap is even more substantial in the more complex E-3 environment, with the PMR-Dueling DQN achieving a reward of 1200 versus much lower rewards of 400, 700, and 900 for Q-learning, DQN, and DDQN, respectively. The consistent superior performance of the PMR-Dueling DQN across both environments demonstrates its ability to learn optimal policies more efficiently. The simplicity of Q-learning limits its performance, while DQN and DDQN show improvements but are still outclassed by the proposed PMR-Dueling DQN’s ability to leverage dueling architecture and Prioritized Experience Replay for sample efficient learning.

In Table 5, the success rates and convergence time of the Q-learning algorithm, the DQN algorithm, the DDQN algorithm, and the PMR-Dueling DQN algorithm, which are used for the path planning task, are compared. Table 5 shows the success rate of reaching the target point and the convergence time of the four algorithms in environment E-2, which only contains static obstacles. The PMR-Dueling DQN algorithm has a 28.2% improvement in success rate compared to the DDQN algorithm, a 35.3% improvement compared to the DQN algorithm, and a 57.9% improvement compared to the Q-learning algorithm. Table 5 also compares the convergence speeds of these four algorithms. It was found that the PMR-Dueling DQN converged to the target fastest out of all approaches, taking 107 min, compared to 268 min for Q-learning, 139 min for DQN, and 116 min for DDQN. Overall, the PMR-Dueling DQN required the least time to obtain the final target point, evidencing faster learning of the task compared to the other algorithms analyzed.

Table 5 also displays the same quantitative evaluation comparison across the four reinforcement learning algorithms but under the more complex environment E-3, which contains both static and dynamic obstacles. The performance of all four algorithms decreases with the inclusion of dynamic obstacles. However, it still shows that the PMR-Dueling DQN has the highest success rate compared to the other three methods with a 38.1% improvement in success rate compared to the DDQN algorithm, a 47.0% improvement over the DQN algorithm, and a 58.2% higher success rate than the Q-learning algorithm. The convergence speeds of the four algorithms on a particular task are also presented in Table 5. The results show that the PMR-Dueling DQN reached convergence most rapidly, attaining the target goal in 122 min. This was faster than Q-learning, which required 276 min to converge; faster than the DQN algorithm, which needed 161 min; and also faster than DDQN, which took 143 min. This demonstrates the superior performance of the PMR-Dueling DQN, which reached the quickest convergence in both dynamic and static versions of the environment. In summary, these results highlight the superior performance of the PMR-Dueling DQN, which displayed quicker convergence and learning speeds on this task per the convergence timing shown in the table.

**Figure 13:**
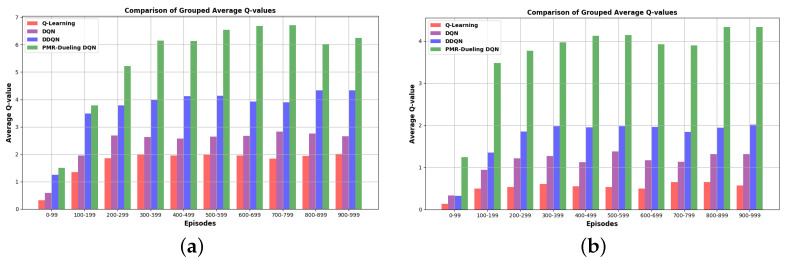
The average Q-values comparison of the four algorithms: (**a**) in environment E-2, which only contains static obstacles, and (**b**) in environment E-3, which contains both static and dynamic obstacles.

The chart shown in Figure 13 is divided into intervals of 100 episodes each, and, for each interval, the average Q-value for each algorithm is displayed as a grouped bar. This visual representation offers insights into how these algorithms perform over time and how their Q-values evolve during training. The bar graphs show that the PMR-Dueling DQN achieves significantly higher maximum average Q-values compared to the other algorithms in both environments. In E-2, it obtains a max Q-value of 6.75 versus 2 for Q-learning, 2.8 for DQN, and 4.36 for DDQN. The advantage is even more substantial in E-3, with the PMR-Dueling DQN reaching 4.34 while Q-learning, DQN, and DDQN only achieve 0.66, 1.39, and 2.03, respectively. The consistently higher Q-values indicate the PMR-Dueling DQN’s superior ability to approximate the optimal action-value function. This leads to more informed action selection and, in turn, better rewards, as evidenced by the reward graphs. The simpler algorithms, such as Q-learning, struggle to obtain high Q-values, limiting their performance. Overall, the PMR-Dueling DQN clearly learns a better Q-function approximation resulting in superior path planning.

## 6. Conclusions

This work demonstrated how deep reinforcement learning combined with novel network design, experience replay, and reward shaping can greatly enhance the performance of mobile robot path planning. These developments are used by the proposed PMR-Dueling DQN algorithm which significantly outperforms conventional Q-learning, DQN, and DDQN techniques in both grid world and Gazebo simulation environments. The higher sampling efficiency, stability, and final policy quality of the PMR-Dueling DQN have been highlighted in evaluations conducted in both a grid world and a realistic Gazebo simulation environment. Compared to sparse binary rewards, the shaped Reward function offers a rich training signal to facilitate faster convergence. Dueling architecture, conversely, separates state and action representations to enable more precise value learning. This is further complemented by Prioritized Experience Replay, which concentrates training on the most informative transitions. This study verifies efficiency in high-fidelity Gazebo environments, bridging the gap between simulation and reality.

While results are promising for simulated settings, further research is needed to evaluate performance in real-world environments with physical robot platforms. A logical next phase of research involves extending the evaluation of our proposed PMR-Dueling DQN approach to physical robots operating in real-world environments. While the simulation environments provide valuable initial development spaces, real-world dynamics and uncertainty will further challenge the generalization capabilities. By deploying onto platforms equipped with sensors for perceiving trajectories and obstacles, such as the Turtlebot with LIDAR and RGB-D cameras, we can rigorously benchmark real-time planning performance across diverse navigation tasks. Success across office building traversals and in outdoor terrain would demonstrate the robustness of the methodology to complex spaces. Transitioning to real-world autonomous navigation remains the ultimate milestone in progressing from simulation. This effort to address interactive dynamics and partial observability would strengthen the proposed technique’s capabilities and maturity towards impactful applications.

## Figures and Tables

**Table 1:** Used Software and Hardware configurations.

Software	Hardware
Python 3.7	16 GB RAM memory
TensorFlow (backend for Keras) https://www.tensorflow.org/	Nvidia GeForce GTX 1050 GPU
Robot Operating System (ros melodic)	Intel Core i5-9300H CPU
Gazebo 9	-

**Table 2:** Hyperparameters and their values in both environment types.

Parameter	For E-1	For E-2 and E-3
Number of episodes	3000	1000
Learning rate	0.01	0.00025
Discount factor	0.9	0.99
Minibatch size	32	128
Replay memory size	10,000	10,000
α	0.5	0.6
β	0.5	0.4

**Table 3:** The final Q-table values with their respective best actions for the PMR-Dueling DQN algorithm.

Status	Action to Up	Action to Down	Action to Right	Action to Left
[3.0,3.0,17.0,17.0]	$1.130\times 10^{-9}$	$4.159\times 10^{-8}$	$1.698\times 10^{-10}$	$9.773\times 10^{-10}$
[3.0,23.0,17.0,37.0]	$1.320\times 10^{-9}$	$3.298\times 10^{-7}$	$1.006\times 10^{-12}$	$2.624\times 10^{-9}$
[23.0,23.0,37.0,37.0]	$1.234\times 10^{-10}$	$2.894\times 10^{-7}$	$-7.449\times 10^{-1}$	$1.956\times 10^{-9}$
[43.0,23.0,57.0,37.0]	$3.917\times 10^{-11}$	$5.689\times 10^{-7}$	$-5.742\times 10^{-1}$	$9.710\times 10^{-10}$
[63.0,23.0,77.0,37.0]	$3.157\times 10^{-11}$	$-6.656\times 10^{-1}$	$1.277\times 10^{-6}$	$1.868\times 10^{-8}$
[83.0,23.0,97.0,37.0]	$4.259\times 10^{-9}$	$6.448\times 10^{-1}$	$-2.860\times 10^{-6}$	$1.546\times 10^{-8}$
[103.0,23.0,117.0,37.0]	$8.304\times 10^{-10}$	$5.073\times 10^{-6}$	$6.161\times 10^{-10}$	$6.654\times 10^{-8}$
[123.0,23.0,137.0,37.0]	$6.029\times 10^{-9}$	$4.260\times 10^{-8}$	$1.325\times 10^{-5}$	$1.388\times 10^{-7}$
[143.0,23.0,157.0,37.0]	$4.530\times 10^{-10}$	$2.704\times 10^{-8}$	$1.460\times 10^{-5}$	$3.583\times 10^{-7}$
[143.0,43.0,157.0,57.0]	$8.547\times 10^{-7}$	$5.570\times 10^{-7}$	$4.352\times 10^{-5}$	$3.976\times 10^{-8}$
[143.0,63.0,157.0,77.0]	$2.156\times 10^{-6}$	$9.055\times 10^{-7}$	$1.116\times 10^{-4}$	$5.704\times 10^{-8}$
[163.0,63.0,177.0,77.0]	$1.889\times 10^{-7}$	$2.250\times 10^{-4}$	$2.762\times 10^{-6}$	$4.763\times 10^{-6}$
[163.0,83.0,177.0,97.0]	$6.753\times 10^{-6}$	$4.379\times 10^{-8}$	$7.159\times 10^{-4}$	$8.159\times 10^{-6}$
[163.0,103.0,177.0,117.0]	$4.346\times 10^{-6}$	$8.272\times 10^{-4}$	$4.272\times 10^{-5}$	$7.689\times 10^{-7}$
[163.0,123.0,177.0,137.0]	$3.355\times 10^{-5}$	$1.447\times 10^{-3}$	$1.354\times 10^{-7}$	$2.073\times 10^{-6}$
[163.0,143.0,177.0,157.0]	$7.454\times 10^{-5}$	$2.501\times 10^{-8}$	$7.130\times 10^{-5}$	$7.825\times 10^{-6}$
[163.0,163.0,177.0,177.0]	$9.339\times 10^{-5}$	$4.254\times 10^{-5}$	$4.254\times 10^{-5}$	$1.258\times 10^{-6}$
[163.0,183.0,177.0,197.0]	$2.264\times 10^{-4}$	$1.271\times 10^{-5}$	$7.072\times 10^{-3}$	$5.077\times 10^{-6}$
[183.0,183.0,197.0,197.0]	$4.483\times 10^{-5}$	$1.283\times 10^{-5}$	$1.138\times 10^{-2}$	$3.193\times 10^{-4}$
[203.0,183.0,217.0,197.0]	$7.260\times 10^{-5}$	$5.061\times 10^{-5}$	$1.798\times 10^{-2}$	$7.168\times 10^{-4}$
[223.0,183.0,237.0,197.0]	$7.499\times 10^{-5}$	$2.680\times 10^{-2}$	$2.042\times 10^{-4}$	$7.128\times 10^{-4}$
[223.0,203.0,237.0,217.0]	$1.380\times 10^{-3}$	$4.175\times 10^{-2}$	$3.859\times 10^{-5}$	$1.057\times 10^{-4}$
[243.0,203.0,257.0,217.0]	$2.553\times 10^{-4}$	$2.806\times 10^{-4}$	$5.385\times 10^{-2}$	$2.373\times 10^{-3}$
[263.0,203.0,277.0,217.0]	$1.580\times 10^{-4}$	$2.287\times 10^{-4}$	$7.279\times 10^{-2}$	$4.591\times 10^{-3}$
[283.0,203.0,297.0,217.0]	$7.453\times 10^{-5}$	$2.299\times 10^{-2}$	$9.690\times 10^{-3}$	$3.823\times 10^{-3}$
[303.0,203.0,317.0,217.0]	$1.127\times 10^{-3}$	$1.251\times 10^{-1}$	$3.131\times 10^{-3}$	$5.609\times 10^{-3}$
[303.0,223.0,317.0,237.0]	$1.384\times 10^{-2}$	$3.561\times 10^{-1}$	$1.566\times 10^{-3}$	$2.770\times 10^{-3}$
[323.0,223.0,337.0,237.0]	$3.099\times 10^{-3}$	$1.901\times 10^{-1}$	$3.058\times 10^{-3}$	$1.593\times 10^{-2}$
[323.0,243.0,337.0,257.0]	$2.888\times 10^{-2}$	$1.002\times 10^{-1}$	$2.254\times 10^{-2}$	$4.244\times 10^{-3}$
[343.0,243.0,357.0,257.0]	$6.546\times 10^{-3}$	$2.624\times 10^{-2}$	$1.565\times 10^{-1}$	$3.689\times 10^{-2}$
[343.0,263.0,357.0,277.0]	$3.308\times 10^{-2}$	$2.820\times 10^{-3}$	$3.011\times 10^{-1}$	$7.740\times 10^{-3}$
[363.0,263.0,377.0,277.0]	$1.417\times 10^{-2}$	$3.412\times 10^{-1}$	$1.013\times 10^{-2}$	$5.315\times 10^{-2}$
[363.0,283.0,377.0,297.0]	$6.754\times 10^{-2}$	$3.832\times 10^{-3}$	$7.472\times 10^{-1}$	$3.249\times 10^{-3}$
[363.0,303.0,377.0,317.0]	$9.205\times 10^{-2}$	$4.283\times 10^{-2}$	$3.097\times 10^{-1}$	$3.811\times 10^{-2}$
[363.0,323.0,377.0,337.0]	$9.063\times 10^{-2}$	$2.581\times 10^{-2}$	$4.773\times 10^{-1}$	$1.766\times 10^{-2}$
[383.0,323.0,397.0,337.0]	$2.752\times 10^{-2}$	$5.310\times 10^{-2}$	$3.690\times 10^{-1}$	$1.023\times 10^{-2}$
[383.0,343.0,397.0,357.0]	$1.106\times 10^{-1}$	$6.341\times 10^{-2}$	$5.903\times 10^{-1}$	$2.969\times 10^{-2}$
[383.0,363.0,397.0,377.0]	$7.694\times 10^{-3}$	$7.639\times 10^{-1}$	$3.576\times 10^{-3}$	$-5.851\times 10^{-2}$
[383.0,383.0,397.0,397.0]	$2.604\times 10^{-3}$	$4.860\times 10^{-1}$	$2.006\times 10^{-2}$	$-2.970\times 10^{-2}$
[383.0,383.0,397.0,397.0]	$2.604\times 10^{-3}$	$4.860\times 10^{-1}$	$2.006\times 10^{-2}$	$-2.970\times 10^{-2}$
[383.0,403.0,397.0,417.0]	$8.078\times 10^{-2}$	$1.000\times 10^{+00}$	$3.603\times 10^{-1}$	$-3.573\times 10^{-1}$

The values in bold indicate the optimal actions selected along the entire path. The value highlighted in red denotes the initial value of the final Q-table. The green highlights the ultimate value of the final goal state, which has a value of ‘+1’.

**Table 4:** Performance Comparison between PMR-Dueling DQN, DDQN, DQN, and Q-learning.

Performance Parameters	PMR-DQN	DDQN	DQN	Q-Learning
Episodes	3000	3000	3000	3000
Shortest path steps	40	43	72	103
Longest path steps	1577	1798	3579	5909
Success rate (%)	94.3	83.7	62.84	37.43

**Table 5:** Comparison of algorithms on different environments.

Method	Success Rate	Convergence Time/min
Algorithm comparisons on environment E-2		
Q-learning	26.7	268
DQN	49.3	139
DDQN	56.4	116
PMR-Dueling DQN	84.6	107
Algorithm comparisons on environment E-3		
Q-learning	21.4	276
DQN	32.6	161
DDQN	41.5	143
PMR-Dueling DQN	79.6	122

The values in bold indicate the performance values for the proposed PMR-Dueling DQN method.

## Data Availability

The data that support the findings of this study are available from the corresponding author upon reasonable request, as they are not publicly available due to privacy reasons.
